# Surface-Dependent Osteoblasts Response to TiO_2_ Nanotubes of Different Crystallinity

**DOI:** 10.3390/nano10020320

**Published:** 2020-02-13

**Authors:** Yuliya Y. Khrunyk, Sergey V. Belikov, Mikhail V. Tsurkan, Ivan V. Vyalykh, Alexandr Y. Markaryan, Maxim S. Karabanalov, Artemii A. Popov, Marcin Wysokowski

**Affiliations:** 1 Ural Federal University, Mira Str. 19, 620002 Yekaterinburg, Russia; s.v.belikov@urfu.ru (S.V.B.); m.s.karabanalov@urfu.ru (M.S.K.); a.a.popov@urfu.ru (A.A.P.); 2 Institute of High-Temperature Electrochemistry of the Ural Branch of the Russian Academy of Sciences, Akademicheskaya Str. 20, 620990 Yekaterinburg, Russia; 3 M.N. Mikheev Institute of Metal Physics of the Ural Branch of the Russian Academy of Sciences, Sofia Kovalevskaya Str. 18, 620219 Yekaterinburg, Russia; 4 Leibniz Institute of Polymer Research Dresden, 01069 Dresden, Germany; tsurkan@ipfdd.de; 5 Max Bergmann Center of Biomaterials Dresden, Hohe Str. 6, 01069 Dresden, Germany; 6 Yekaterinburg Research Institute of Viral Infections, Rospotrebnadzor, Letnyaya Str. 23, 620030 Yekaterinburg, Russia; vyalykhivan@yandex.ru (I.V.V.); alek.marckarian2010@yandex.ru (A.Y.M.); 7 Institute of Chemical Technology and Engineering, Faculty of Chemical Technology, Poznan University of Technology, Berdychowo 4, 60965 Poznan, Poland

**Keywords:** TiO_2_ nanotubes, anodization, amorphous, anatase, osseointegration, gene expression, implants

## Abstract

One of the major challenges of implantology is to design nanoscale modifications of titanium implant surfaces inducing osseointegration. The aim of this study was to investigate the behavior of rat osteoblasts cultured on anodized TiO_2_ nanotubes of different crystallinity (amorphous and anatase phase) up to 24 days. TiO_2_ nanotubes were fabricated on VT1–0 titanium foil via a two-step anodization at 20 V using NH_4_F as an electrolyte. Anatase-phase samples were prepared by heat treatment at 500 °C for 1 h. VT1–0 samples with flat surfaces were used as controls. Primary rat osteoblasts were seeded over experimental surfaces for several incubation times. Scanning electron microscopy (SEM) was used to analyze tested surfaces and cell morphology. Cell adhesion and proliferation were investigated by cell counting. Osteogenic differentiation of cells was evaluated by qPCR of runt-related transcription factor 2 (RUNX2), osteopontin (OPN), integrin binding sialoprotein (IBSP), alkaline phosphatase (ALP) and osteocalcin (OCN). Cell adhesion and proliferation, cell morphology and the expression of osteogenic markers were affected by TiO_2_ nanotube layered substrates of amorphous and anatase crystallinity. In comparison with flat titanium, along with increased cell adhesion and cell growth a large portion of osteoblasts grown on the both nanostructured surfaces exhibited an osteocyte-like morphology as early as 48 h of culture. Moreover, the expression of all tested osteogenic markers in cells cultured on amorphous and anatase TiO_2_ nanotubes was upregulated at least at one of the analyzed time points. To summarize, we demonstrated that amorphous and anodized TiO_2_ layered substrates are highly biocompatible with rat osteoblasts and that the surface modification with about 1500 nm length nanotubes of 35 ± 4 (amorphous phase) and 41 ± 8 nm (anatase phase) in diameter is sufficient to induce their osteogenic differentiation. Such results are significant to the engineering of coating strategies for orthopedic implants aimed to establish a more efficient bone to implant contact and enhance bone repair.

## 1. Introduction

Today one of the main challenges of implantology is to achieve a direct contact between bone and the implant without the formation of a fibrous capsule. Bone is a complex tissue constantly undergoing dynamic biologic remodeling: while mature bone is resorbed by osteoclasts, new bone is generated by osteoblasts, thus maintaining healthy homeostasis of bone [1]. Although many advances in biomaterials and tissue engineering have been reached, repair of a critical-sized non-healable bone defects (exceeding 5 mm) still remains a challenge, however. The use of autograft tissue (taken from patient) causes the morbidity due to a second surgical site, while the application of allograft (another person’s tissue) poses the risk of potential disease transmission and tissue rejection [1,2]. Due to such significant drawbacks of autograft and allograft applications it is crucial to search for efficient biomimetic materials [3,4,5,6] and structures [7,8,9], suitable for skeletal repair without inherent problems.

Owing to their good biocompatibility, low density, satisfactory mechanical strength and superior corrosion resistance titanium (Ti) and its alloys are widely used materials in orthopedic and dental surgery [10,11]. Notably, implants biocompatibility and integration into the bone depend on their surface topography [12], hence the ability of titanium to be topographically modified at the nanometer scale is another useful feature of this material. The potential of physical and topological cues as an osteogenic trigger has been studied by different research groups. The effect of nanoscale topological surface modifications has been studied on various cell lines including human mesenchymal stem cells (MSCs) [13,14], rat MSCs [15], human osteoblast cell lines [13,16,17], neural crest-derived stem cells [18], osteosarcoma-derived MG63 [19] and Saos-2 [20], MC3T3-E1 mouse osteoblasts [21,22]. The most recent technologies to produce nano-texturized surface modifications include a relatively simple and economically feasible anodic oxidation method generating highly ordered coatings of TiO_2_ nanotube arrays [23,24,25]. Such surfaces exhibited an increase in hydrophilic properties and surface energy [26,27]. Moreover, they demonstrated enhanced osteoconductivity by providing anchorage sites for osteoblast filopodia extensions, which leads to early differentiation and significant proliferation of cells [16]. By modifying the conditions of the anodizing process, i.e., the applied potential, current density, pH value, anodization time, temperature and concentration of fluorine ion, different morphological (diameter, height and surface area) parameters of TiO_2_ nanotubes can be achieved [25,28,29]. Though much less studies have been dedicated to the role of titanium crystallinity (amorphous versus anatase structures) in osseointegrative properties of titania nanotubes, it was shown, however, that the crystallization, usually conducted via heat treatment [30], results in enhanced mechanical strength of as-anodized amorphous titania nanotubes [31] and increased hydrophilicity, which eventually might improve cell adhesion and proliferation [32,33].

The aim of this study was to investigate the behavior of primary rat osteoblasts cultured on anodized TiO_2_ nanotubes of different crystallinity (amorphous and anatase phase) up to 24 days. By applying anodic oxidation to VT1–0 titanium, specific nanoscale parameters (i.e., inner diameter (*Di*), height (*H*) and surface area (*Sa*)) have been fabricated yielding amorphous substrates (AM) with 31 ≤ *Di* ≤ 39 nm, 1485 ≤ *H* ≤ 1565 nm and 747 ≤ *Sa* ≤ 1829 nm^2^, followed by annealing at 500 °C for 2 h to produce anatase substrates (AN) with 33 ≤ *Di* ≤ 49 nm, 1496 ≤ *H* ≤ 1546 nm and 1162 ≤ *Sa* ≤ 2806 nm^2^. The effect of AM, AN and flat-surfaced VT1–0 Ti substrates on the adhesion, proliferation, morphology and the expression of osteogenic markers, i.e., runt-related transcription factor 2 (RUNX2), osteopontin (OPN), integrin binding sialoprotein (IBSP), alkaline phosphatase (ALP) and osteocalcin (OCN), was evaluated. To our knowledge, this is the first study to give a comprehensive view on osseointegrative effects of TiO_2_ nanotubes of amorphous and anatase crystallinity (produced on VT1–0 titanium) with a range of specific nanoscale surface parameters. 

## 2. Materials and Methods 

### 2.1. Substrate Preparation and Fabricating Nanotubes

Squares (40 mm × 40 mm) were cut from 0.125 mm thick titanium foil VT1–0, the chemical composition of which meets State Standard 19807–91 and is presented in Table 1. After polishing by emery paper squares were polished using 15 µm alumina slurry to produce a smooth surface. After polishing, they were rinsed thoroughly and sonicated in isopropyl alcohol for about 20 min. 

Anodic titanium dioxide layers on Ti samples were prepared via a two-step anodization in electrolyte solution containing NH_4_F (0.5 g, Sigma-Aldrich, Steinheim, Germany) and H_2_O (50 mL) at 20 °C. The process was carried out at 20 V in a two-electrode fluoroplastic cell, with polished Ti squares as anodes and a platinum plate as a cathode. The duration of the first and second anodizing steps were 1 h and 15 min, respectively. All used chemicals were of analytical grade.

The post-anodization heat treatment was conducted by heating titania nanotube foils at 500 °C for 1 h in air using a hot plate. The titania nanotube samples were placed directly onto the hot plate, which was preheated and maintained at 500 °C to ensure a constant temperature. They were covered by inverted glass beaker to minimize convective heat loss from the top surface. Next, the titania nanotube samples were removed from the hot plate and cooled to room temperature in air. All the samples used for in vitro experiments employing cell cultures were sterilized in a stem autoclave at 120 °C for 30 min. To analyze the characteristics of substrates after anodization (of amorphous and anatase crystallinity), SIAMS Photolab software (SIAMS Carlsbad, CA, USA) was employed.

### 2.2. Cell Cultures

To obtain cell culture of primary rat osteoblasts (herein referred as osteoblasts), we used a modified method based on several osteoblast isolation protocols [34,35]. Briefly, calvarial bone fragments were grinded and then digested for 30 min with an enzymatic cocktail containing 0.1% collagenase IV (Gibco, Waltham, MA, USA) and 0.25% trypsin-EDTA (Ethylene diamine tetraacetic acid) (Sigma-Aldrich, Steinheim, Germany). Next, bone explants were cultured in Dulbecco’s modified Eagle’s medium (DMEM)/F-12HAM (Sigma) supplemented with 20% fetal calf serum (FCS), 2 mM L-glutamine, 100 U/mL penicillin-streptomycin and 1% non-essential amino acids (all reagents from Sigma, herein referred as DMEM medium) in 25 cm^2^ culture flasks (TPP Techno Plastic Products AG, Trasadingen, Switzerland) in a humidified 5% CO_2_ atmosphere (Esco, Singapore). Osteoblast migration from rat bone fragments (Figure S1) was observed on days 10–14. Cells displayed rapid proliferation starting from passage 3 (Figure S1). To run cell culture assays, osteoblasts (fourth passage) were plated in DMEM medium on tested surfaces (flat titanium surface, surface with titanium nanotubes of amorphous and anatase crystallinity and cell culture plastic (TPP Techno Plastic Products AG, Trasadingen, Switzerland)) at a density of 10^4^/cm^2^ without additional (biochemical) osteogenic cues. Cells were further cultivated at 37 °C and 5% CO_2_ in a humidified incubator. 

### 2.3. Scanning Electron Microscopy

Characterization of the tested titanium surfaces and cells, cultured on different substrates, was performed using Auriga CrossBeam (Carl Zeiss Microscopy, GmbH, Jena, Germany) scanning electron microscope at an acceleration voltage of 1 kV and a beam current of 60 pA. Images were acquired using both the InLens secondary electron detector and the energy selected backscattered electron detector. Data was collected at 5000–50,000× magnification. To visualize cells, cultures on different substrates (flat Ti, AM and AN), samples were washed with phosphate-buffered saline (PBS) and fixed in 2.5% glutaraldehyde (Sigma) in 0.1 M PBS for 24 h (pH = 7.4, room temperature). Next, samples were dehydrated in a graded series of ethanol (50%, 60%, 70%, 80%, 90% and 99.8%) for 10 min each and left in 99.8% alcohol until they were dried. 

### 2.4. XRD Analysis

X-ray diffraction (XRD) analysis was conducted employing CuKα_1,2_ radiation on a Shimadzu-7000 diffractometer (Shimadzu Corp., Kyoto, Japan) with the Bragg–Brentano recording geometry. XRD patterns were measured via step-scan mode at Δ(2θ) = 0.02° in the 2θ angular range from 10 to 100° with a long exposure time at each step. The surface and side views of synthesized samples were examined on SIGMA VP (Carl Zeiss) scanning electron microscope under high vacuum using InLens detector. Obtained SEM images were analyzed with Clinker C7 software by SIAMS (SIAMS Software, Carlsbad, CA, USA).

### 2.5. RNA Isolation, Primer Design and qRT-PCR

Expression of runt-related transcription factor 2 (RUNX2), osteopontin (OPN), integrin binding sialoprotein (IBSP), alkaline phosphatase (ALP) and osteocalcin (OCN) genes was quantified in cells, cultured on flat Ti, AM (amorphous), AN (anatase) and cell culture plastic for 1, 4 and 24 days. Total RNA from osteoblasts cultivated on tested substrates was isolated at days 1, 3 and 24 using the Trizol reagent (Sigma-Aldrich). Oligonucleotide primers were designed using Clone Manager 9 (Sci-Ed Software, Westminster, Colorado, CO, USA see Table 2). The concentration of purified RNA was quantified using a NanoDrop (NanoDrop Technologies, Wilmington, DE, USA) and 1 μg of total RNA was used to synthesize cDNA templates by oligo(dT) priming using the Superscript First-Strand cDNA Synthesis System (ThermoFisher Scientific, Waltham, MA, USA). qRT-PCR was performed with the ABI Prism 7500 Sequence Detection System (Applied Biosystems; 40 cycles; melting for 15 s at 95 °C; annealing and extending for 60 s at 60 °C) using Power SYBR Green PCR Master Mix (Invitrogen, Waltham, MA, USA). The threshold cycle (Ct) values were calculated from amplification pots. The ΔCt value for each sample was obtained by subtracting the Ct values of a housekeeping gene, glyceraldehyde phosphate dehydrogenase (GAPDH). Fold changes compared with the control (flat Ti) were determined by raising 2 to the power −ΔCt as outlined in the protocol of Applied Biosystems. The number of samples included in each experiment for control and treatment conditions were three, and each experiment was carried out at least three times. The data are represented as mean ± SEM for triplicate values. Statistical analyses were performed by Student’s *t*-test. 

### 2.6. Cell Adhesion and Proliferation

Cell adhesion was investigated 1 day after seeding the cells on four tested substrates (flat Ti, AM, AN and cell culture plastic) and proliferation was investigated after days 2 and 4. Tested substrates were rinsed in PBS to remove any non-adherent cells. The remaining cells were fixed with formaldehyde, stained with Hoechst 33258 dye (Sigma) and counted under a fluorescent microscope (Zeiss), 5 random fields were counted per substrate. All experiments were conducted in triplicate and repeated at least three separate times.

### 2.7. Resazurin Assay

Cell proliferation 24 h post seeding on four tested substrates (flat Ti, AM, AN and cell culture plastic) was analyzed employing resazurin conversion assay. Following washing in PBS tested substrates were incubated with a 1:10 volume of resazurin (Sigma, Germany) working solution (0.025% in Dulbecco’s PBS at 37 °C up to 150 min. Fluorescence (ex/em = 540 nm/590 nm) was measured every 30 min spectroscopically (FLUOstar Omega, BMG, Ortenberg, Germany) and corrected to background control. The assay was conducted in triplicate.

### 2.8. Protein Quantification

To verify resazurin assay data, cell density on tested substrates (flat Ti, AM, AN and cell culture plastic) was analyzed by quantifying protein content using sulforhodamine B (SRB) colorimetric assay. Following resazurin assay, cells were washed with PBS and stored in 99.9% ethanol at −20 °C until being used for protein quantification. Briefly, ethanol was removed, cells were washed with tap water, air-dried and stained with 0.4% (w/v) SRB in 1% (v/v) acetic acid for 30 min. After the dye was removed by washing 3–4 times with 1% (v/v) acetic acid, stained protein was dissolved in 10 mM Tris-based solution for spectrophotometric measurement at 550 nm.

### 2.9. Alizarin Red Staining

Alizarin red staining was conducted after 24 d of culture. Mineralization assay was carried out on cells, co-cultured with Ti, AM and AN substrates in DMEM medium. After the culture medium was removed cell layers were washed with PBS and stained with Alizarin Red S (2%, Merck, Darmstadt, Germany) for 2 min. Cells were visualized employing Zeiss Axio Observer A1 inverted microscope. 

### 2.10. Statistical Analysis

At least three independent experiments were conducted, each in triplicate. Numerical data were analyzed using standard analysis of variance (ANOVA) followed by two-tailed unpaired Student’s *t* test. A *p* < 0.05 was considered statistically significant. 

## 3. Results

### 3.1. Surface Morphology of Titania Nanotubes Layered Substrates of Amorphous and Anatase Crystallinity

**Figure 1:**
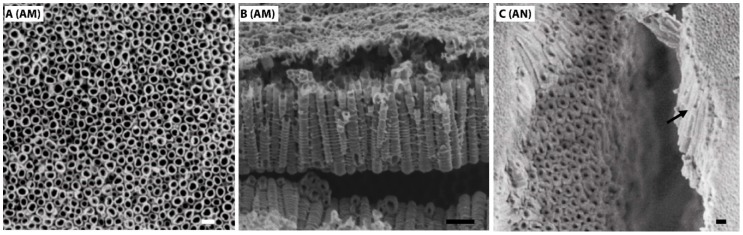
Surface SEM images of TiO_2_ nanotubes fabricated at 20 V ((**A**), amorphous crystallinity), (**B**) shows lateral view of amorphous TiO_2_ nanotubes, anatase nanotubes after annealing at 500 °C for 2 h are shown in (**C**), lateral view is indicated by an arrow. Scale bar shows 100 nm.

The surface and cross-sectional SEM images of the TiO_2_ nanotubes, fabricated on Ti VT1–0 under 20 V in ammonium fluoride (AM) and then annealed at 500 °C for 2 h (AN) are shown in Figure 1. XRD patterns demonstrating initial titanium foil and nanotubular TiO_2_ layer grown via anodization are shown in Figure S2. 

The surface morphology reveals a homogenous distribution of highly ordered, vertically aligned, hollow titania nanotubes (TNTs) in AM and AN (Figure 1A,C). Flat Ti substrates with a native TiO_2_ oxidation layer on the VT1–0 surface (having analogous chemical composition to the AM and AN substrates, see Materials and Methods) were used as comparison substrates.

In this study, nanotube inner diameter, surface area and height are denoted as *Di*, *Sa* and *H*, respectively. TiO_2_ nanotubular layers were fabricated with average sizes of *Di* equal to 35 ± 4 and 41 ± 8 nm for AM and AN substrates, respectively. Likewise, the annealing of TNTs-layered substrates also led to increase in the *Sa* values (see Table 3), i.e., 1288 ± 541 nm^2^ and 1984 ± 822 nm^2^ for AM and AN, respectively, however, it did not affect the TNTs height, which was equal to 1525 ± 40 nm and 1521 ± 25 nm for AM and AN, respectively.

**Figure 2:**
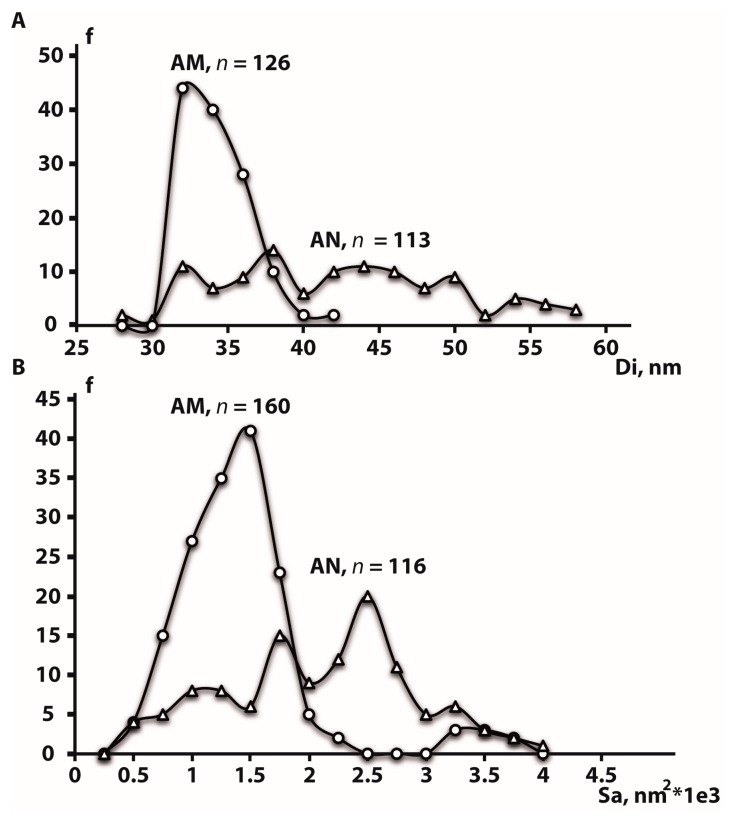
The plots showing the distribution of inner diameter (**A**) and surface area (**B**) for amorphous (AM) and anatase (AN) surfaces. Titania nanotubes count frequency, inner diameter and surface area are denoted as f, Di and Sa, respectively.

Figure 2A,B demonstrates poly-dispersed values of *Di* and *Sa* of AM and AN surfaces. *Di* distribution in AM surfaces is characterized by a single distinct peak showing that the most of nanotubes (67%) are characterized by *Di* of 32–34 nm. Though annealing led to an increase in the average *Di*, AN surfaces revealed a much wider *Di* frequency dispersal without any main peak (Figure 2A). While *Di* of 42 nm was a maximum for AM surfaces, 45% of AN TNTs had a *Di* ranging from 44 to 58 nm indicating that almost a half of AM TNTs has undergone an increase in their *Di* during the annealing at 500 °C. A similar trend was observed when AM and AN surface areas were compared (Figure 2B). 79% of AM surfaces showed *Sa* ranging from 1000 to 1750 nm^2^. On the other hand, a maximum frequency for *Sa* in AN surfaces was shifted to 2500 nm^2^, and 58% of AN TNTs demonstrated *Sa* within the range of 1750–2750 nm^2^. 

### 3.2. Comparative Study of Osteoblast Adhesion on Tested Surfaces on Days 1, 2 and 4

**Figure 3:**
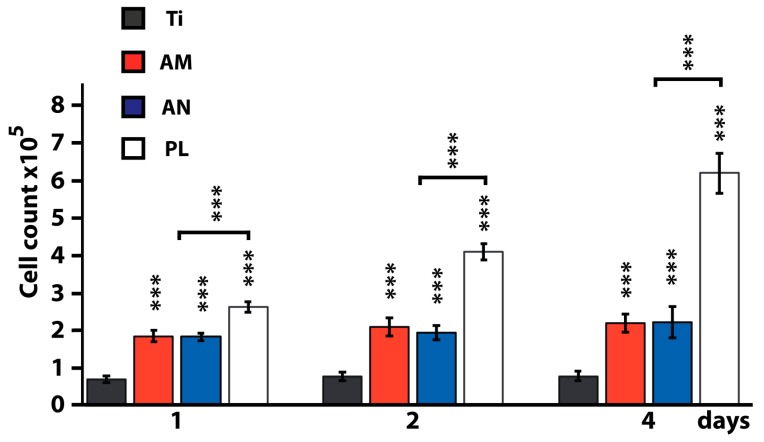
Primary rat osteoblasts adhesion and proliferation on flat titanium (Ti), amorphous (AM), anatase (AN) titania nanotubes (TNTs) substrates and cell culture plastic (PL) on day 1, 2 and 4. *** *p* < 0.01 for tested substrates versus Ti and for AM and AN versus PL.

Attachment of osteoblasts to potential implant surfaces enables them to spread and differentiate. To test the effect of physical and mechanical properties of TNTs-layered surfaces on cell adhesion and cell proliferation, we seeded osteoblasts on AM and AN and compared analyzed parameters with those from flat Ti and cell culture plastic (Figure 3). The highest cell adhesion and proliferation was observed on cell culture plastic. However, in comparison with flat Ti substrates, the number of cells on AM and AN surfaces was significantly increased (*p* < 0.01) at all three time points, i.e., 161%, 163% and 182% increase in the number of osteoblasts was observed at 1, 2 and 4 days of culture, respectively (Figure 3). Regarding the crystallinity of TNTs our data indicated no statistically significant differences between AM and AN at any of the observed time points. Additionally, 24 h post seeding the cells on tested substrates, resazurin conversion assay was conducted to analyze mitochondrial activity which serves as an indirect measurement of cell proliferation (Figure S3). Within the analyzed time range (30–150 min) significantly increased fluorescence was observed in cells cultured on TNTs-layered substrates compared to flat Ti. The highest metabolic activity was shown by cells cultured on cell culture plastic (Figure S3), which is in agreement with cell proliferation results on day 1. These data were further confirmed by protein content analysis (Figure S4). 

### 3.3. Morphological Features of Cells Cultured on Tested Surfaces

**Figure 4:**
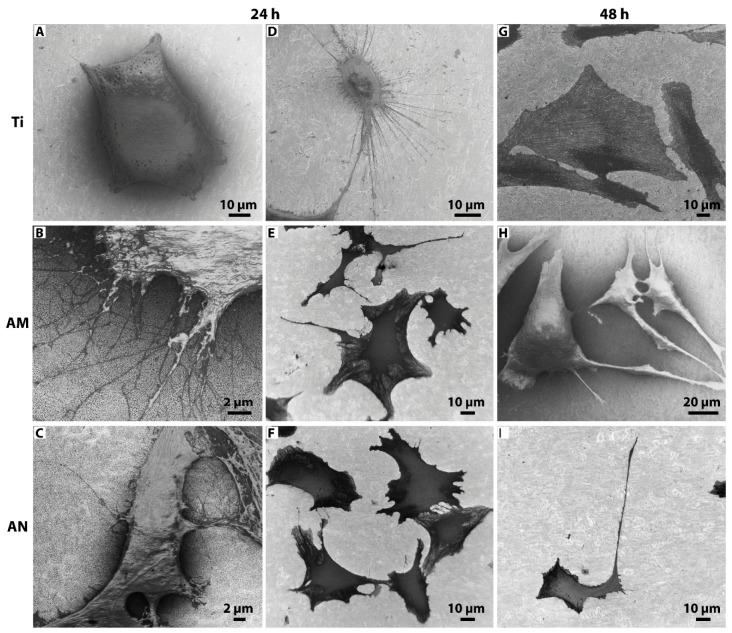
SEM micrographs of osteoblasts seeded on (**A**,**D**,**G**) flat Ti; (**B**,**E**,**H**) amorphous and (**C**,**F**,**I**) anatase TNTs layers after 24 and 48 h of culture.

**Figure 5:**
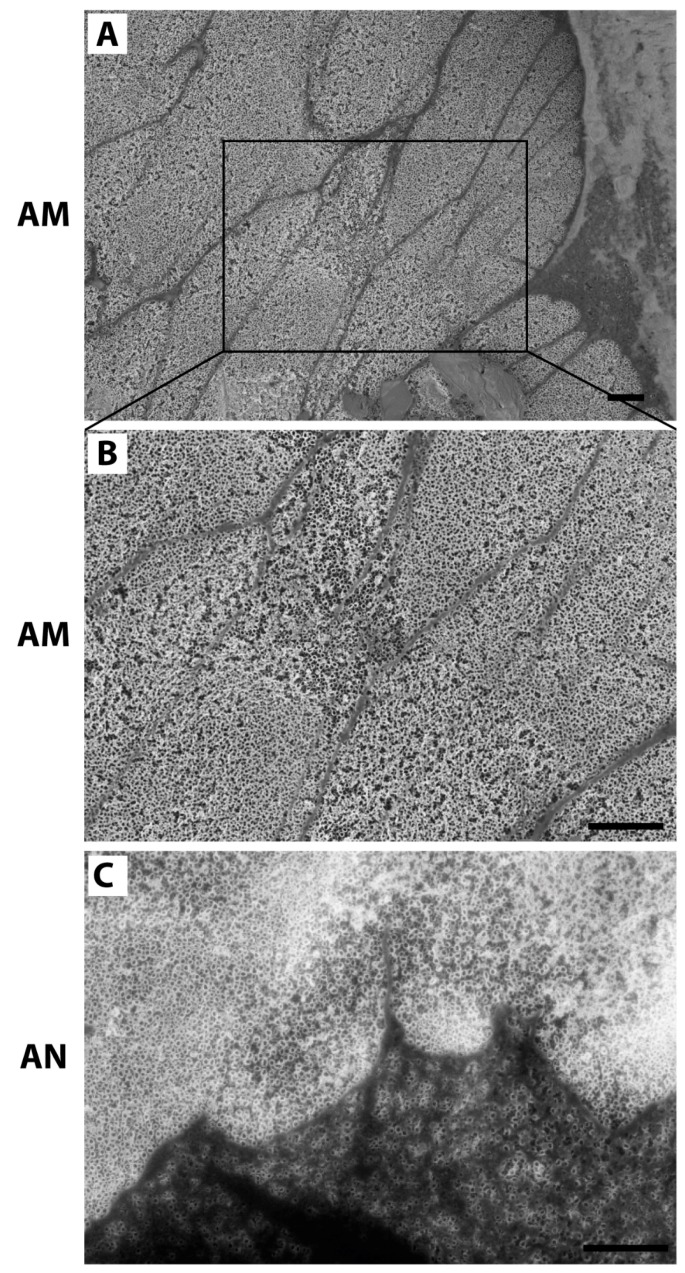
Cell filopodia travelling over nanotubes of amorphous (**A,B**) and anatase (**C**) phases, scale bar = 1 µm.

Morphological analysis of cells grown on tested substrates (flat Ti, AM and AN) for 24 and 48 h was performed by SEM (Figure 4). SEM images of cells cultured on experimental substrates showed that after 24 and 48 h of incubation the shape of cells on flat Ti and TNTs-layered substrates (AM and AN) were extremely different. After 24 h of culture on flat Ti osteoblasts displayed minimal spreading lacking distinct filopodia extensions (Figure 4A) or a rather round small cell body with numerous «ray-like» filopodia (Figure 4D). In contrast, cells grown on AM and AN surfaces were spread freely and developed extensive filopodia and lamellipodia (Figure 4B,C,E,F), which might have facilitated cell anchorage to the nanotubular structures. After 48 h of culture, cells on flat Ti exhibited mostly a polygonal shape (Figure 4G), whereas a great portion of osteoblasts cultured on AM and AN demonstrated extraordinary long unidirectional lamellipodia extensions, which exceeded the size of cell body more than twice indicating osteogenic differentiation (Figure 4H,I). The morphology of the underneath nanotubular layer was visible at higher magnification as shown in Figure 5. As is shown by SEM images (Figure 5), the diameter of osteoblast filopodia exceeded TNTs diameter and abundant filopodia were spread over large areas of nanotube layers. Morphological changes of cells, cultured on AM and AN substrates, pointed to surface-directed differentiation of osteoblasts into osteocyte-like cells, which was further analyzed by qPCR.

### 3.4. Osteogenic Differentiation of Osteoblasts Cultured on Tested Surfaces

**Figure 6:**
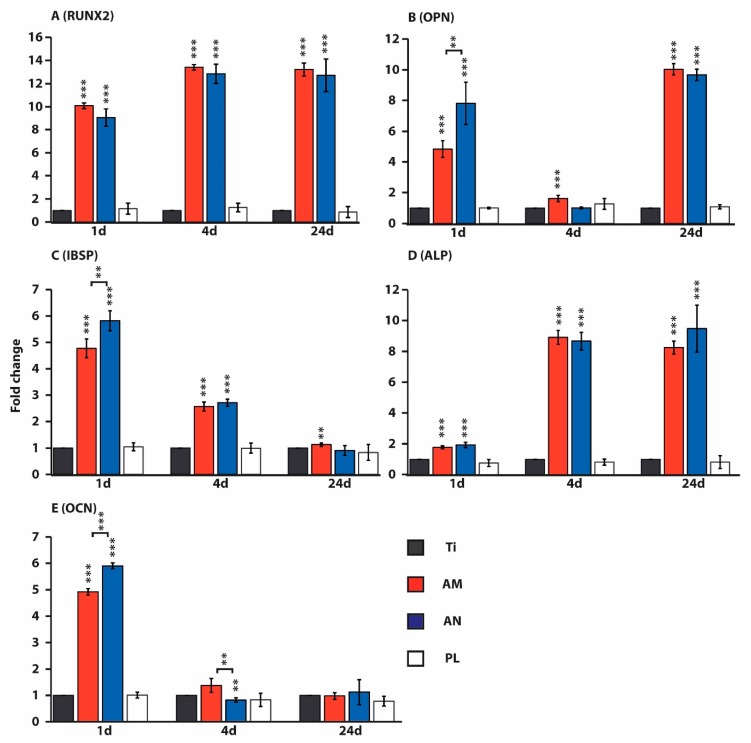
Effects of nanostructured titanium surfaces on mRNA expression of osteogenic genes in osteoblasts, cultured for 1, 4 and 24 days on flat titanium (Ti), anodized titanium of different crystallinity (amorphous (AM) and anatase (AN)) and cell culture plastic (PL). qRT-PCR of mRNA of runt-related transcription factor 2 (RUNX2, **A**), osteopontin (OPN, **B**), integrin binding sialoprotein (IBSP, **C**), alkaline phosphatase (ALP, **D**) and osteocalcin (OCN, **E**). ** *p* < 0.05, *** *p* < 0.01 for tested substrates versus Ti and for AM vs. AN.

Due to the prominent role of runt-related transcription factor 2 (RUNX2), osteopontin (OPN), integrin binding sialoprotein (IBSP), alkaline phosphatase (ALP) and osteocalcin (OCN) in osteogenic differentiation we analyzed their quantity by qRT-PCR. RNA was isolated from osteoblasts, cultured on tested substrates (flat Ti, AM, AN, plastic) for 1, 4 and 24 days mRNA expression is presented as a fold change compared to the expression on flat Ti surface (Figure 6). The expression of RUNX2 in cells, cultured on AN substrates for 1 day, was significantly up-regulated (9 fold) and further increased on days 4 and 24 (12 fold; Figure 6A). Osteoblasts, grown on AN surfaces for 1 day, demonstrated elevated level of OPN mRNA, which was significantly higher on AN (7 fold) compared to AM surfaces (4 fold). On day 4, the expression of OPN on the AN surface was on the same level as flat Ti, however, it was slightly up-regulated on AM surfaces (1.5 fold). On day 24, in osteoblasts cultured on both AM and AN substrates, OPN transcript levels were significantly raised again (9 fold, Figure 6B). Likewise, significantly elevated levels of IBSP transcripts were detected in cells, cultured on AN substrates for 1 day, however, the expression of IBSP on AN surface was higher (5 fold on AN vs. 4 fold on AM surface). Over day 4 and day 24 the IBSP expression gradually decreased, moreover, on day 24 the level of IBSP transcripts in cells, cultured on AN surface, did not differ from the data, observed on flat Ti (Figure 6C). In contrast, the expression of ALP in cells, cultured on AN surfaces for 1 day, was only slightly upregulated (1.8 fold), whereas it was significantly up-regulated on day 4 (9 fold) and remained to be expressed at the same level compared to the flat Ti substrate (Figure 6D). Alike to the expression patterns of OPN and IBSP, a significant induction of OCN expression was detected in cells, cultured on AN substrates on day 1, and its level for AN slightly exceeded the quantity of OCN transcripts on AM samples (6 fold on AN vs. 5 fold on AM surface). Over days 4 and 24, the levels of OCN transcripts did not exceed OCN expression levels for flat Ti, moreover, on day 4 cells cultured on AN surface displayed a slight down-regulation of OCN (Figure 6E). In cells, cultured on plastic, transcript levels of all the tested osteogenic markers was not significantly different from the values, obtained for flat Ti. Therefore, in contrast to cells, cultured on flat Ti and plastic, osteoblasts grown on TNTs demonstrated the upregulation of all the tested osteogenic markers at least on one of the recorded time points (Figure 6).

**Figure 7:**
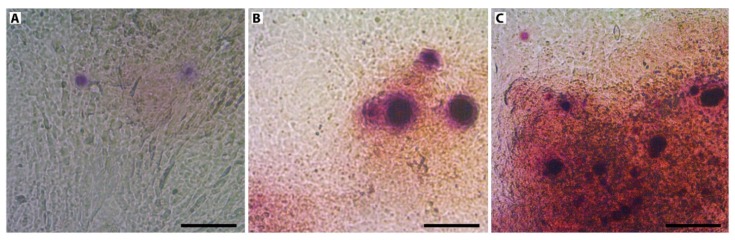
Alizarin red staining of cells cultured on flat titanium (**A**), amorphous (**B**) and anatase TiO_2_ layered substrates (**C**) in non-osteogenic medium after 24 d of culture. Scale bar: 100 µm.

In order to test matrix mineralization, alizarin red S staining was conducted in standard non-osteogenic conditions on day 24 (Figure 7). Staining for mineralization was negative for cells cultured on Ti, whereas positive staining was observed on AM and AN substrates, which was in line with observed osteogenic gene expression profiles.

## 4. Discussion

The layers of highly ordered, vertically aligned titania nanotubes arrays were formed by electrochemical anodization, a method producing nanostructures on the surface of metal-based implants such as Ti, Ti-based alloys, tantalum and zirconium [25]. The diameter of TNTs depends on the current density as it changes the electrochemical etching rate: the increase in current density results in larger TNTs diameter [25]. In our experimental set-up we employed NH_4_F as fluoride ion source, the conductivity of which (70 mS/cm) is higher in comparison with electrolytes based on NaF (50 mS/cm) and a weak HF acid (17 mS/cm) [38]. It also should be noted that an aqueous electrolyte (used in our study) requires much less processing time and the voltage to form TNTs with the same diameter as the ones generated employing organic electrolyte [25].

In this study, average sizes of TNT *Di*, *Sa* and *H* were equal to 35 ± 4 and 41 ± 8 nm, 1288 ± 541 and 1984 ± 822 nm^2^ and 1525 ± 40 and 1521 ± 25 nm for AM and AN, respectively. The post-annealing increase in TNTs pore size is in line with the observations of Yang et al. [39] who demonstrated that the annealing of TNTs with an average diameter of 40 nm resulted in the increase of the average TNTs diameter to 50 nm without affecting uniform morphology of TNTs-layered surfaces. This may be attributed to the dehydration reaction, which occurs during the phase transformation from amorphous to anatase [39,40].

A significant increase in the number of osteoblasts grown on AM and AN substrates (161%, 182% and 179% on days 1, 4 and 24) in comparison to flat titanium surface is in line with the data demonstrated by several other researchers with different types of nanostructured surfaces. 

In particular, Zhang et al. [22] demonstrated a significant difference in mouse osteoblasts proliferation on the surface of flat Ti and TNTs arrays (diameter of which was in the range from 150 to 470 nm) at all analyzed time points. Likewise, the differences in cell number and proliferation rate between the amorphous and anatase phased TNTs did not exhibit statistical significance. Using the same cell culture of mouse osteoblasts, MC3T3-E1, Oh et al. [41] reported a significant acceleration of cell adhesion and proliferation on TNTs (*Di* 70 nm; *H* 250 nm): after 48 h of incubation, the number of cells on TNTs substrates were about 300–400% higher in comparison with flat Ti surfaces. The authors, however, showed a reduced level of cell number, cultured on AM TNTs as compared to AN TNTs arrays. Furthermore, employing human osteoblastic precursor cell line, Das et al. [16] showed that the number of cells grown on TNTs (*Di* 50 nm) was higher than flat Ti surfaces at all tested time points with the most significant increase at day 16. Finally, a 40% increase in the number of rat MSCs, cultured on TNTs (*d* 80 nm; *H* 400 nm) in comparison with flat Ti surfaces was recorded after 7 days of culture [15].

SEM images of osteoblasts grown on Ti (24 h) demonstrated cells lacking filopodia or displaying a rather small round cell body with numerous filopodia, whereas at 48 h after seeding they acquired a polygonal shape. On the other hand, cells cultured on both TNT-layered surfaces (AM and AN) were spread freely and displayed extensive filopodia and lamellipodia (24 h), most of which developed extremely long unidirectional lamellipodia extensions, observed already at 48 h after seeding. Cellular spreading and adhesion depending on TiO_2_ nanoscale environments has been studied on various cell lines. In particular, using surfaces of vertically aligned TNTs with six different diameters Park et al. [42] demonstrated that on nanotubes with a diameter larger than 30 nm (i.e., 50, 70 and 100 nm) MSCs did not spread properly and showed unstable filopodia extensions. Moreover, the researchers revealed that primary human osteoblast-like cells (hOBs) spread out normally on smaller size nanotubes (15 nm) forming lamellipodia and wide thick filopodia, which was not observed on the TNTs surfaces with a diameter of 100 nm. In contrast, cultivation of cells on the latter led to significant cell apoptosis. Similar data were obtained in a comparative analysis employing Ti substrates, layered with titania nanopillars of different height (15, 55 and 100 nm, [19]) showing that lower (15 nm) pillar structures led to increased cell spreading, cytoskeletal development and bone matrix nodule formation. In the study conducted by Cowden et al. (2019) human adipose-derived stem cells were cultured on TiO_2_-layered substrates with the inner diameter ranging from 70 to 160 nm. In comparison to flat Ti cells seeded on nanotubes demonstrated elongated morphology with long filopodia with the most elongated cells observed on surfaces with nanotubes of 160 nm [43].

According to Park et al. [42], a surface geometry with a lateral spacing of about 15 nm corresponds to the size of integrin heads and hence will be recognized by MSCs and bone remodeling cells. It also should be noted that the mechanism of cell adhesion is driven by promoting the adsorption of selected proteins (such as a high-molecular-weight nanometer-sized glycoprotein fibronectin), which are important for integrin-mediated communication between cells and surface. Oh et al. [44] assumed that flat Ti had a lower population of fibronectin aggregates due to its lower hydrophilicity in comparison with higher-surface-area TNTs surfaces, the latter was shown by water droplet contact angle tests [16,33,45,46]. Moreover, the efficiency of fibronectin adhesion to TNTs surfaces varies depending on the size of TNTs pores. In a comparative assay employing nanotubes of different pore size Oh et al. [44] revealed that TNTs of 30 and 50 nm in diameter accumulated a much higher population of protein aggregates on the top surface in comparison to TNTs with a larger diameter (70 and 100 nm). Apparently protein aggregates, which have a size of about 30 nm, are too small to adhere on 70- or 100-nm diameter TNTs with much larger open pore spaces [44]. Indeed, to the hypothesis of Oh et al. [44], human MSCs are more easily adhered to the surface with TNTs ≤ 50 nm as attached to the nanopores serum proteins played the role of extracellular matrix (ECM) proteins. In our study, AM and AN TNTs had a diameter of ≤ 50 nm, which, according to the studies of Oh et al. [44], fits to a threshold point of nanotube diameter size, suitable for fibronectin attachment, followed by increased level of cell adhesion. In contrast, osteoblasts cultured on flat Ti apparently struggled to adhere to the surface (Figure 4D) and hence developed extensively long filopodia in order to find ECM proteins.

Likewise, using human osteoblasts, grown on 50 nm-diameter TNTs surfaces, Das et al. [16] revealed 2–3 fold increase in cell attachment and spreading in comparison to flat Ti samples. This is also consistent with the results of Yu et al. [28] analyzing the adhesive and osteogenic impact of anatase titania nanotubes of different diameters (20, 50, 70, 100 and 120 nm). Using MC3T3-E1 preosteoblasts the authors observed that filopodia spread well over nanotube layers with 20, 50 and 70 nm in diameter, whereas they seemed to avoid TNTs with a larger diameter, 100 and 120 nm. It should be mentioned, however, that not all the studies supported the hypothesis of advantageous ≤ 50 nm TNTs diameter. According to Popat et al. [15], rat-derived MSCs, cultured on 80 nm-diameter TNTs substrates, demonstrated higher cell adhesion, viability and proliferation up to 7 days of cultures in comparison with flat Ti. Such cells also demonstrated greater ALP activity along with calcium and phosphorus deposition [15]. Moreover, in contrast to Park et al. [42], a comparative study employing MC3T3-E1 mouse osteoblasts, cultured on anatase-phase TNTs with average diameters of 30, 50, 70 and 100 nm, resulted in a different pattern of cell response [21]. Indeed, according to the trend, observed by Brammer et al. [21], the increase in TNTs diameter was accompanied by the increase in elongation/stretching of cell bodies, ALP levels and bone-forming ability. In particular, TNTs with the largest tested diameters (100 nm) were reported to have the greatest potential for bone implant materials. Finally, Zhang et al. [22] used MC3T3-E1 mouse osteoblasts to test the effect of TNTs with larger diameter (150, 260, 360, 470 and 570 nm). It was shown that the peak of proliferation rate was observed on 470 nm-diameter TNTs (both AM and AN), whereas the highest ALP activity was detected in cells, grown on TNTs (both AM and AN) with 150 nm in diameter.

Filopodia spread over TNTs act as anchor points exploring the surface for optimal environmental cues so that more stable focal contacts could be established. Though some studies revealed that filopodia penetrated into the nanotubes, which led to increase in cell adhesion [15,47], we did not observe filopodia extending towards the inside of TNTs. According to our data (Figure 5), the diameter of osteoblast filopodia exceeded TNTs diameter and numerous filopodia could spread over large areas of nanotube layers. This was in line with the results, provided by Yu et al. [28] demonstrating that the diameter of MC3T3-E1 filopodia was larger than diameters of nanotubes, equal to 20, 50 and 70 nm. Interestingly, though nanotubes with the diameter of 100 and 120 nm were wide enough for MC3T3-E1 filopodia to penetrate them, the filopodia mostly spread between two closely placed nanotubes as if avoiding to spread towards the inside of TNTs.

The results of gene expression analysis (see Figure 6) pointed to the upregulation of all the tested osteogenic markers in cells cultured on AM and AN at least on one of the recorded time points (1, 4 and 24 days). A significant decrease in the expression of OPN, IBSP and OCN (on the 1 day) on AM in comparison with AN might be due to the differences in TNTs parameters, i.e., AM substrates contained a portion of TNTs with almost twice larger pore sizes (Figure 2). Perhaps osteoblast performance on AM substrates was slightly inhibited by the residual fluorine ions, incorporated in TNTs after anodization.

Our study is in line with the findings of Lavenus et al. [13] and Schuermann et al. [18], demonstrating that surfaces with about 30 nm pore diameter are able to induce osteogenic differentiation of human MSCs and human neural crest-derived stem cells, respectively. In particular, human MSCs, cultured on titanium substrates with nanopores of 33 nm in diameter for 12 days, showed upregulation of osteogenic genes, such as RUNX2 and OCN [13]. Osteoblasts, employed in our experiments, displayed a strike increase in OCN expression only on day 1, however, which was also different from its expression pattern, demonstrated by Schuermann et al. [18]. While cultivating human neural crest-derived stem cells on TNTs with about 30 nm pores, Schuermann et al. [18] demonstrated an increased expression level of osteocalcin on day 12, which was further elevated on day 21. Relatively high expression levels of osteogenic markers (ALP, RUNX2, OPN and OCN) were observed by Seo et al. [46] conducting experiments on rat MSCs cultured on AN titanium samples after non-thermal atmospheric pressure plasma jet treatment. Interestingly, Oh et al. [44] showed that cultivation of human MSCs on TNTs of 70 and 100 nm in diameter leads to the up-regulation of osteogenic markers (ALP, OPN and OCN), whereas nanotubes with a diameter of 30 and 50 nm showed no osteoinductive effects. On the other hand, Schuermann et al. [18] reported that cultivation of neural crest-derived stem cells on 100 nm pores resulted in increased apoptosis and had no osteoinductive effects. Elevated levels of RUNX2 also, reported for nanopores of 33 and 193 nm in diameter, were not shown for samples with 149 nm in diameter [13], implying that the osteogenic differentiation of cells, induced by topographic cues, is not a direct function of a pore size. Though diameters of AM and AN TNTs in our study are comparable to the ones employed in the study of Lavenus et al. [13] (*d* = 33.4 ± 0.7 nm, *H* = 5.0–5.7 nm), the height of TNTs fabricated in this research was 300 times higher. It should be mentioned that TNTs height used in our study fits to the size of collagen (200 nm long, [48]), another important ECM protein. Apparently, ideal geometric parameters of nanostructures remain to be established.

To further test the effect of TNT-layered surfaces on cell differentiation, Alizarin Red staining was carried out on day 24. In contrast to osteoblasts, grown on flat titanium substrates, mineralization was visualized on cells cultured on both AM and AN surfaces without osteogenic factors indicating that TNT decorated surfaces fostered osteogenic differentiation of cells. This data is in line with the studies of Lavenus et al. [13] showing osteogenic differentiation of human MSCs grown on TNT-layered substrates with nanotubes of 30 and 150 nm in diameter.

## 5. Conclusions

TiO_2_ nanotubes of different crystallinity (amorphous and anatase) were successfully fabricated on titanium surfaces via a two-step anodization process at 20 V employing NH4F as a fluoride ion source. After the heat treatment, internal diameter and surface area of nanotubes increased. Osteoblasts, cultured on both tested surfaces showed elevated adhesion and proliferation up to 24 days of culture. Osteogenic differentiation of cells grown on AM and AN demonstrated by SEM was accompanied by elevated transcripts of osteogenic markers. The expression of all tested genes (RUNX2, OPN, IBSP, ALP and OCN) were of the same pattern for osteoblasts from AM and AN substrates being upregulated at least at one of the analyzed time points. We assumed that increased adhesion, growth and osteogenic differentiation of cells, cultured on AM and AN surfaces in comparison to flat titanium was mainly due to the nanotopography as significant differences between amorphous and anatase surfaces regarding cell performance were shown only for gene expression at day 1 of culture. In sum, the cultivation of osteoblasts on AM and AN TNTs led to osteogenic differentiation without the need of additional biochemical cues. This in vitro study provides an additional body of evidence to the research on biocompatible and osseointegrative properties of nanotubes fabricated on titanium and its alloys suggesting that TNTs-layered surfaces have a big potential to be used in the manufacturing of therapeutic orthopedic implants.

## Figures and Tables

**Table 1:** Chemical composition of titanium samples, employed for anodization, w.t. %.

Fe	C	Si	N	Ti	O	H	Impurities
≤0.25	≤0.07	≤0.1	≤0.04	99.2–99.7	≤0.12	≤0.01	0.3

**Table 2:** Primers, used for qRT-PCR.

Primer	Sequence (5′-3′)	Accession Number
OPN-YK-fw	GCCGAGAAGCCGGATGCAAT	AB001382.1
OPN-YK-rv	AGGCTGGCTTTGGAACTCGC	
IBSP-YK-fw	AGGGGCATGGCTATGAAGGCT	BC127506.1
IBSP-YK-rv	GGCCGCTACAAACGGAAGCA	
OCN-YK-fw	GGGCCTTTGCTTTCCATATT	M23637.1
OCN-YK-rv	CAGTGGCATTAACCAACACG	
Runx2-fw *	GGCCTTCAAGGTTGTAGCCC	XM_017596552.1
Runx2-rv *	CCCGGCCATGACGGTA	
ALP-fw **	AGGCAGGATTGACCACGG	NM_013059.1
ALP-YK-rv	GCTCACCATGGGAGCCAGAC	
GADPH-YK-fw	AAACCCATCACCATCTTCCA	XM_017593963.1
GADPH-YK-rv	GTGGTTCACACCCATCACAA	

* from Reyes et al. [36], ** from Selvamurugan et al. [37].

**Table 3:** Physical surface features of the amorphous and anatase substrates.

Surface	Inner Diameter (nm)	Surface Area (nm^2^)	Height (nm)
Amorphous	35 ± 4	1288 ± 541	1525 ± 40
Anatase	41 ± 8	1984 ± 822	1521 ± 25

## Supplementary Materials

The following are available online at https://www.mdpi.com/2079-4991/10/2/320/s1, Figure S1: Primary rat osteoblast cell culture from Vistar rat fetal calvaria fragments. Osteoblastic cells migrating from a bone fragment, day 10 (A); cell population of polygonal and spindle-like shape, day 12 (B); Diploid karyotype of primary rat osteoblast cell culture, passage 5, Figure S2. XRD pattern of TiO2 layer. XRD pattern of TiO_2_ layer with the diffuse reflection maximum marked by an arrow. Above XRD pattern of the initial titanium foil is shown for comparison. Figure S3. Resazurin assay 24 h post seeding. Resazurin assay conducted on primary rat osteoblasts cultured on flat titanium (Ti), amorphous (AM) and anatase (AN) TiO_2_ layered substrates and cell culture plastic (PL) 24 h post seeding. Reduction in resazurin fluorescence was observed every 30 min up to 150 min. Figure S4. Sulforhodamine B (SRB) assay. SRB absorbance indicating protein content which is indirect measurement of cell proliferation. Ti, AM, AN and PL demonstrate results for cells grown on flat titanium, amorhous and anatase TiO_2_ layered substrates and cell culture plastic, respectively.
