# Supplementary figures and images for: Surface-Dependent Osteoblasts Response to TiO2 Nanotubes of Different Crystallinity
Source: Nanomaterials (Basel). 2020 Feb 13;10(2):320. doi: 10.3390/nano10020320 (PMC7075131; doi:10.3390/nano10020320)

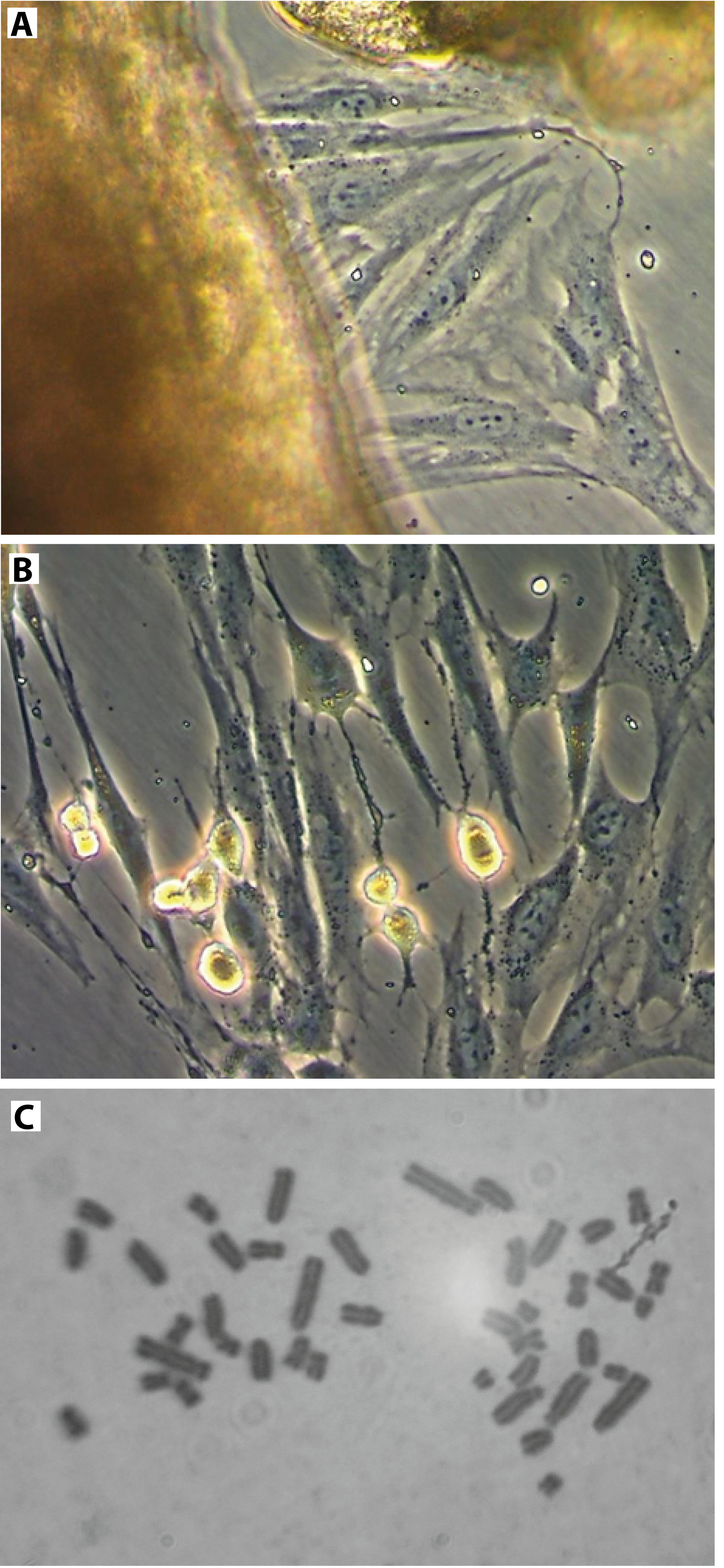

Supplement: Supplementary file 1 [file nanomaterials-10-00320-s001.zip › FigureS1.tif]

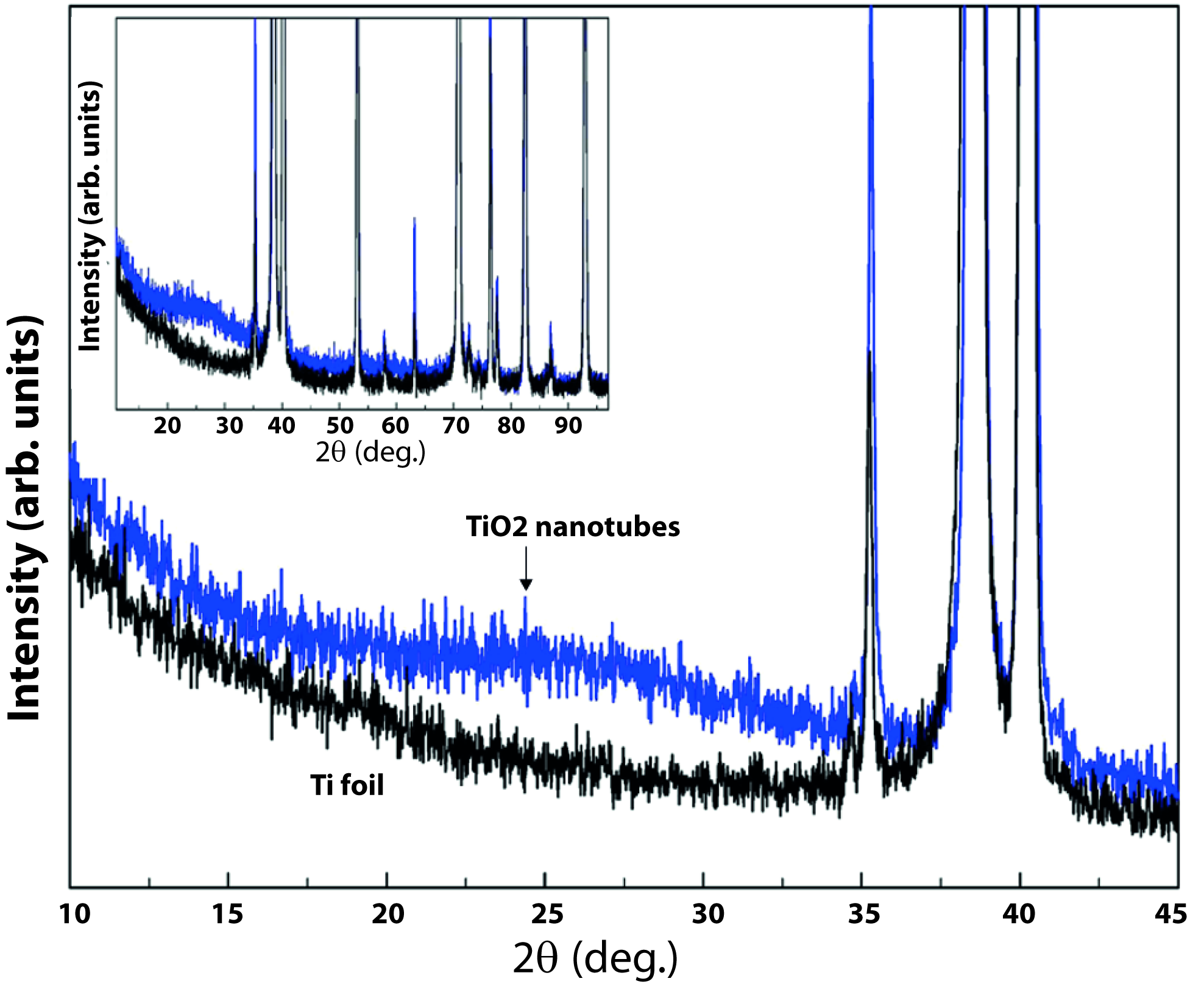

Supplement: Supplementary file 1 [file nanomaterials-10-00320-s001.zip › FigureS2.tif]

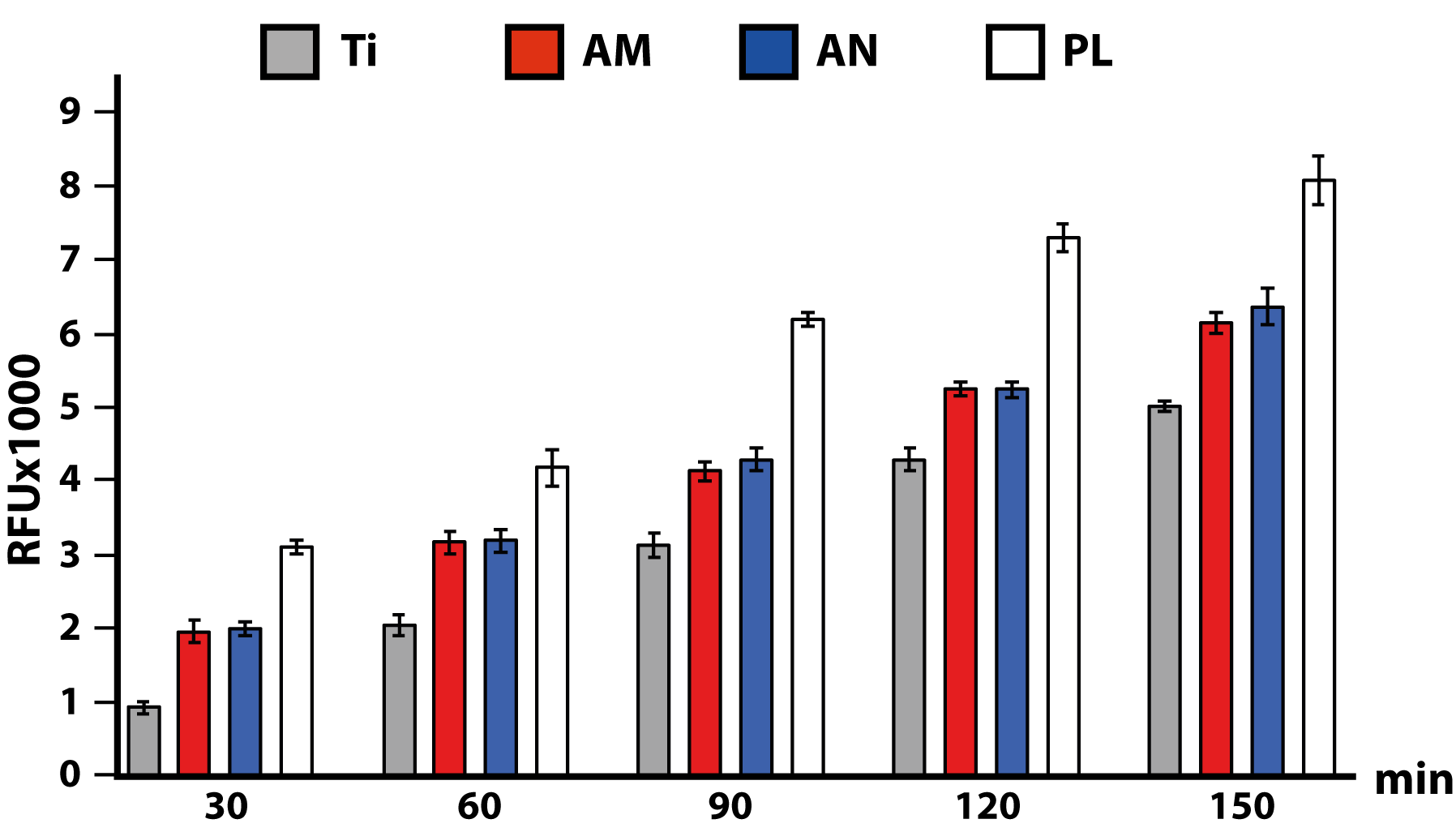

Supplement: Supplementary file 1 [file nanomaterials-10-00320-s001.zip › FigureS3.tif]

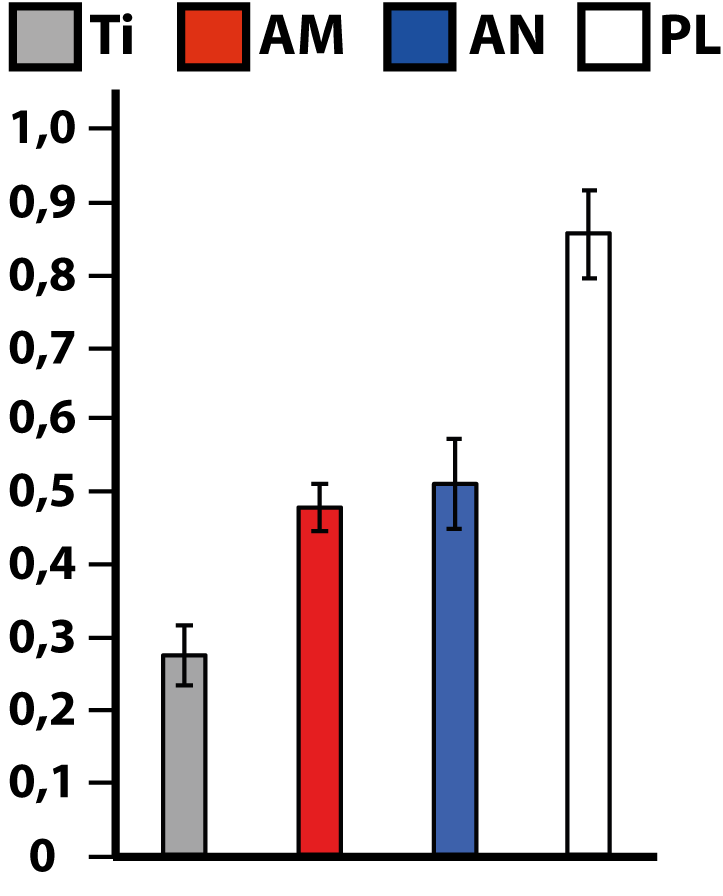

Supplement: Supplementary file 1 [file nanomaterials-10-00320-s001.zip › FigureS4.tif]
